# Tiny Cells, Big Clues: Unveiling Infantile Pyknocytosis Through a Case Series

**DOI:** 10.7759/cureus.93272

**Published:** 2025-09-26

**Authors:** Fathima Amani Mohammed Nowfal, Sura Ahmed, Mahmoud Alhussain Radaideh

**Affiliations:** 1 Pediatrics, Al Jalila Children's Speciality Hospital, Dubai, ARE; 2 Pediatrics, Hatta Hospital, Hatta, ARE

**Keywords:** cardiac defects, hemolytic anemia, neonatal jaundice, pulmonary hypertention, pyknocytes, recombinant human erythropoietin (rhepo)

## Abstract

Infantile pyknocytosis is a rare and benign hematologic disorder with an unknown etiology that may initially present as a critical neonatal condition. Although it clinically appears as neonatal jaundice and Coombs-negative anemia, the diagnosis of infantile pyknocytosis is primarily based on identifying pyknocytes in the peripheral blood smear, along with excluding other common causes of hemolytic anemia.

By presenting case studies of neonates with jaundice and findings suggestive of infantile pyknocytosis, this study underscores the importance of recognizing this uncommon condition in the evaluation of neonatal hemolytic anemia of uncertain etiology. Timely diagnosis and appropriate management can lead to favorable outcomes, highlighting the need for increased awareness among healthcare providers.

## Introduction

Neonatal anemia is defined as a hemoglobin or hematocrit concentration that is more than two standard deviations below the mean for postnatal age. Common causes of anemia in neonates and infants include physiologic anemia and anemia of prematurity. Pathological causes can be categorized into three types: anemia due to blood loss, decreased production, and increased destruction of erythrocytes [1]. Hemolytic anemia in the newborn period occurs as a result of erythrocyte rupture, commonly attributed to well-known causes such as antibody-mediated reactions (ABO incompatibility, Rh incompatibility, or transplacental transfer of maternal anti-red blood cell antibodies), erythrocyte membrane defects (hereditary spherocytosis, hereditary elliptocytosis), and enzyme deficiencies (G6PD deficiency, pyruvate kinase deficiency) [2].

Since 1959, a rare cause of hemolytic anemia in neonates has been described by Tuffy et al., who discovered peculiar red blood cells with irregular margins and rare short projections on peripheral blood smear analysis, which they termed pyknocytes [3]. Since then, infantile pyknocytosis has been recognized to account for 10% of neonatal anemia with an unidentified etiology [4].

Although the cause of this condition is unknown, there are several common factors: neonatal presentation with Coombs-negative jaundice and anemia, diagnosis made exclusively by the presence of more than 5% pyknocytes on peripheral blood smear, and positive results from treatments such as phototherapy, erythrocyte transfusion, and recombinant human erythropoietin, leading to complete remission with no residual effects [5].

## Case presentation

Case 1

A female term newborn, delivered at 38 weeks of gestation to a mother with no prenatal care, presented to our facility with hemolysis at birth. Despite a reassuring physical examination, blood investigations revealed a hemoglobin level of 13.9 g/dL, with a blood film demonstrating features of hemolysis, including irregular contracted RBCs and polychromasia. Leukocyte count and morphology were within normal limits, and platelet levels were adequate on the smear. The mother’s blood group was B positive, while the neonate's was O positive. The direct antiglobulin test (DAT) was negative, and the total bilirubin level at 48 hours of life was 10 mg/dL, increasing to 12.5 mg/dL at 105 hours. Given the reassuring findings and the bilirubin levels being below the phototherapy threshold for the neonate's age, the baby was discharged.

**Figure 1:**
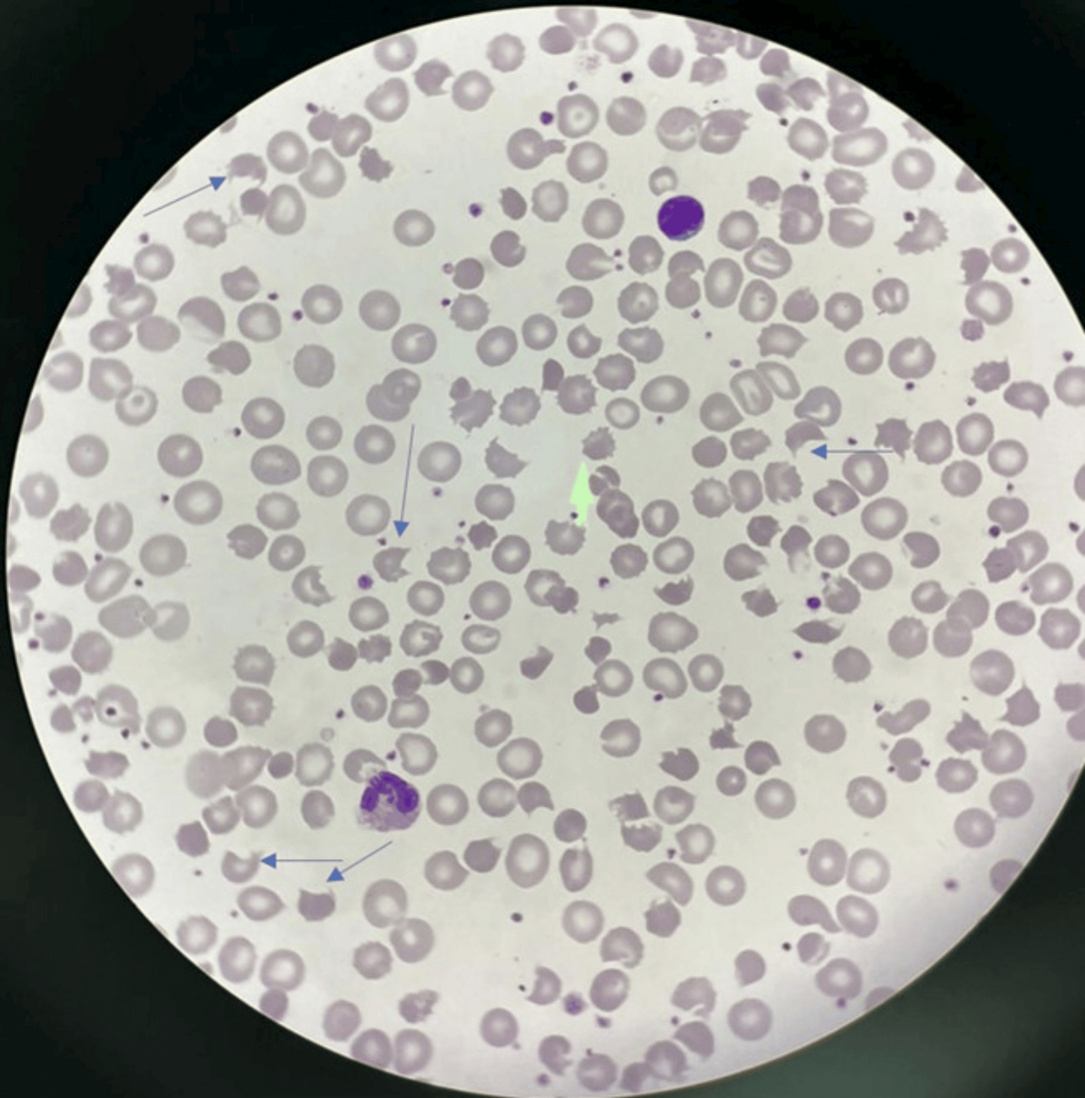
Blood film of case 1: Peripheral smear shows fragmented red blood cells, including burr cells and irregularly contracted RBCs. Blue arrows highlight pyknocytes: small, dense, irregularly shaped erythrocytes. WBCs show neutrophilic leukocytosis, and platelets are increased, suggesting a reactive picture consistent with hemolysis.

However, on the ninth day of life, the baby was readmitted to the medical facility due to jaundice and an elevated bilirubin level of 24 mg/dL, necessitating intensive phototherapy. Despite the absence of risk factors for hemolytic jaundice, as confirmed by negative family history and neonatal screening, the baby experienced a decrease in hemoglobin from 9.3 g/dL upon admission to 8.1 g/dL the following day. Subsequent blood film analysis revealed morphology highly suggestive of infantile pyknocytosis (Figure 1). She received a packed red blood cell (PRBC) transfusion, significantly improving hemoglobin levels to 11.2 g/dL. After a six-day hospital stay, her bilirubin level decreased to 9.8 mg/dL, and she was discharged.

**Figure 2:**
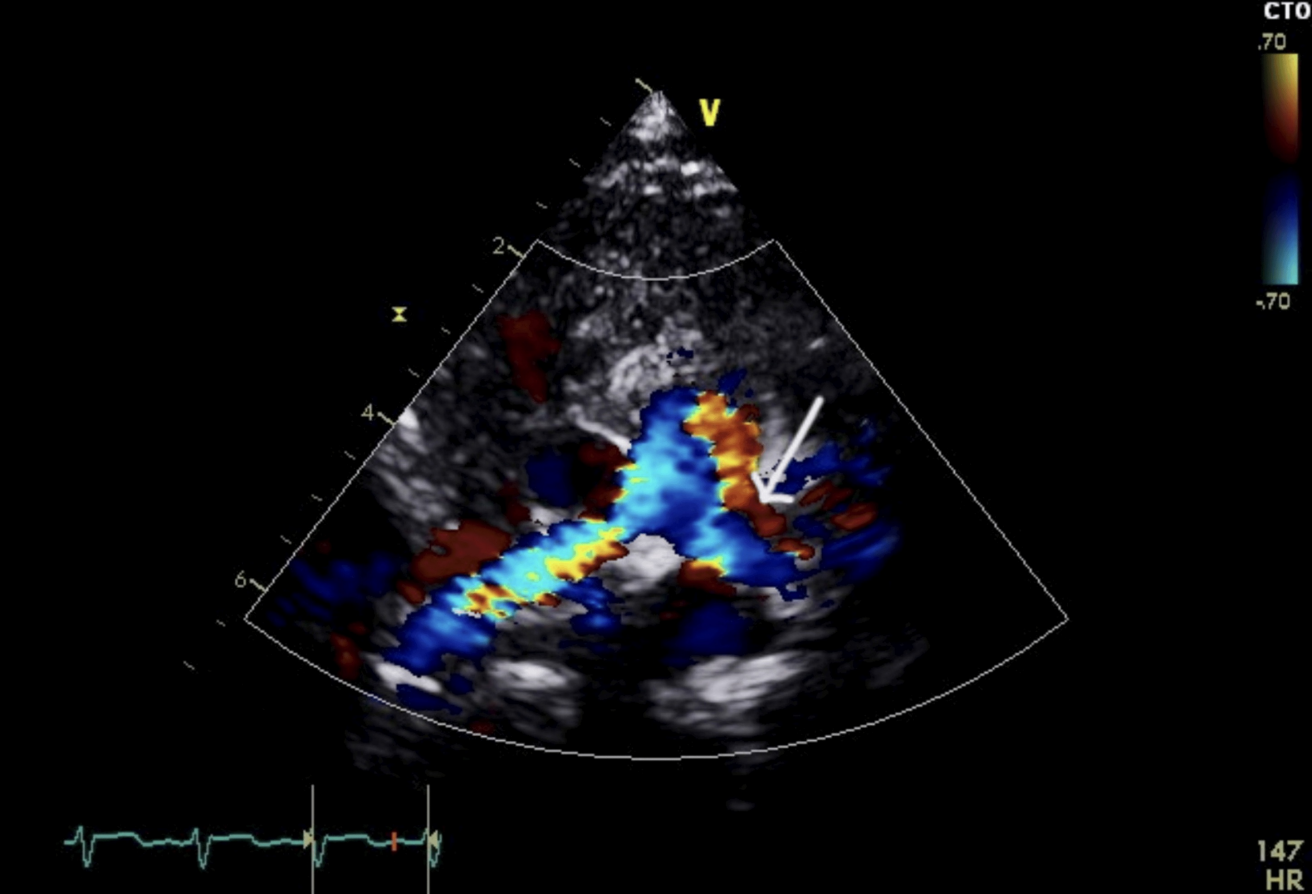
Echocardiogram image of case 1 demonstrating a significant patent ductus arteriosus (PDA) characterized by a bidirectional shunt, predominantly oriented from left to right.

**Figure 3:**
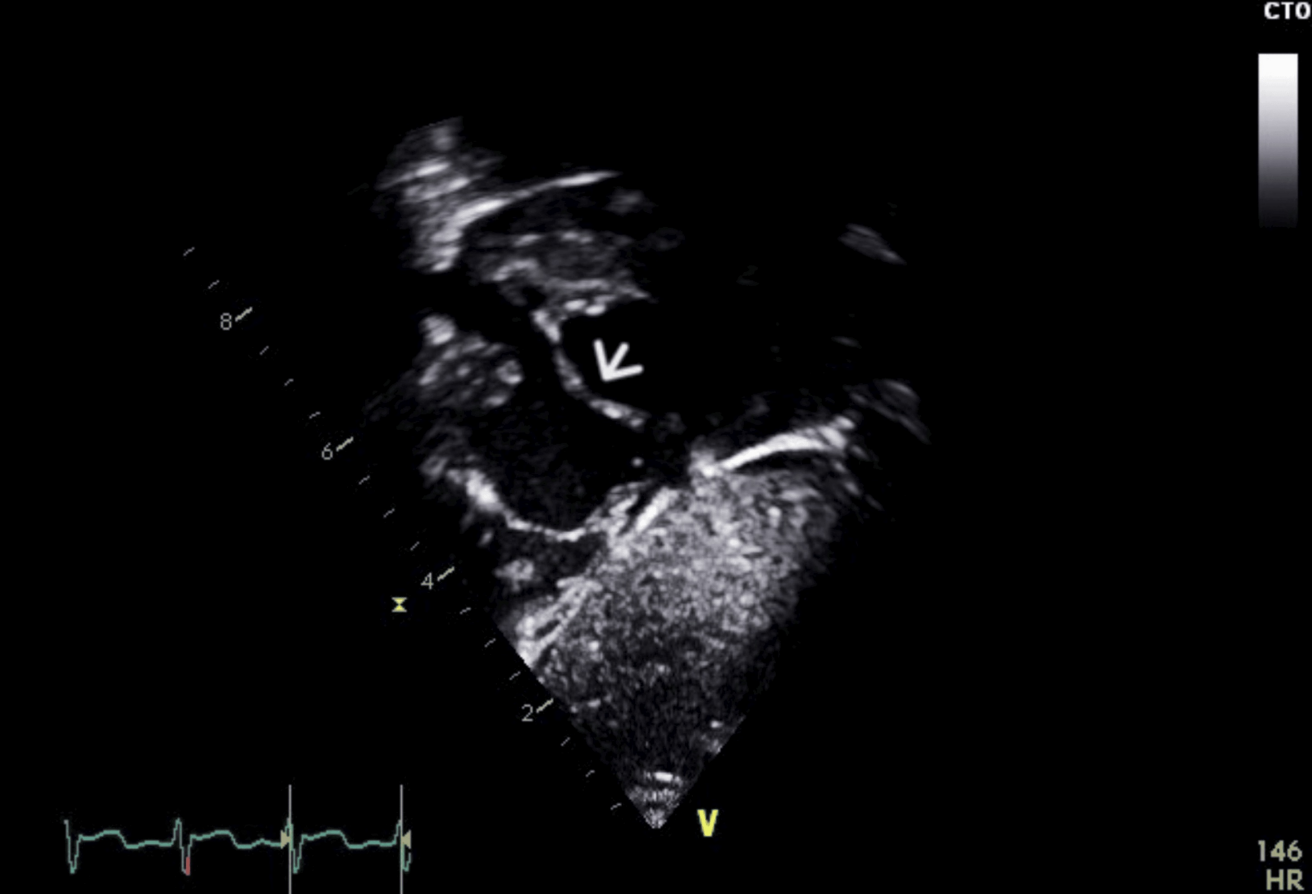
ECHO image of case 1 depicting a 4 mm secundum atrial septal defect.

**Figure 4:**
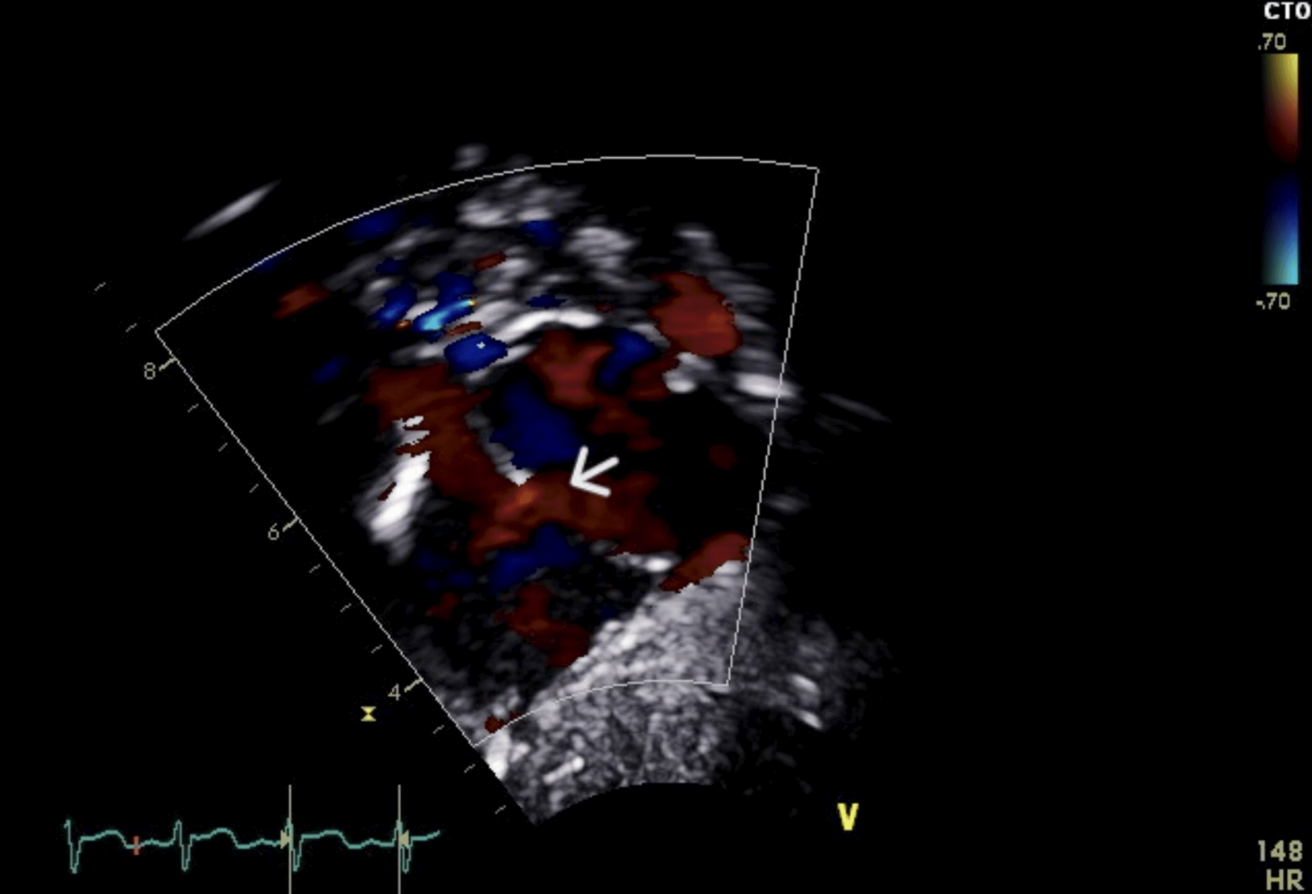
Doppler imaging of case one shows a small atrial septal defect with a left-to-right shunt.

At the age of four weeks, the patient was readmitted to the hospital due to respiratory distress following an enterovirus bronchiolitis infection. Along with the respiratory distress, an incidental systolic cardiac murmur was observed, as well as signs of pallor and hepatomegaly. Laboratory investigations revealed a hemoglobin level of 6.8 g/dL, necessitating a PRBC transfusion. This intervention increased her hemoglobin level to 10.5 g/dL. Further evaluation was conducted to assess the possibility of impending heart failure due to the murmur and hepatomegaly. An echocardiogram (ECHO) revealed a large patent ductus arteriosus (PDA) with a bidirectional shunt, primarily left-to-right, along with signs of volume overload (Figure 2). Additionally, there was a secundum atrial septal defect (ASD) measuring 4 mm in diameter (Figures 3 and 4) and evidence of tricuspid regurgitation, indicating increased pulmonary artery pressure and features suggestive of pulmonary hypertension. As a result, the patient was started on anti-failure medications, including Lasix, spironolactone, and captopril. Her condition was closely monitored, and her hemoglobin level remained stable during follow-up visits.

Case 2

An 18-day-old female infant, born at 36 weeks of gestation via normal vaginal delivery to a mother with unremarkable antenatal scans, serologies, and family history, was admitted to our facility with jaundice first observed at two days of age. The neonate required intensive phototherapy due to a rapid increase in bilirubin levels from 9.2 mg/dL to 14.7 mg/dL over 13 hours. The baby’s blood group was A positive, while the mother's blood group was O positive, and both the DAT and newborn screening results were within normal limits.

For social reasons, the parents elected to discharge the infant against medical advice (DAMA) and sought care at another facility, where the baby received phototherapy for five days before being discharged. Ten days after discharge, she was brought back to our facility due to a urinary tract infection that led to recurrence of jaundice, necessitating further phototherapy. On admission, the baby presented with a bilirubin level of 16.8 mg/dL, a hemoglobin level of 10.5 g/dL, and a reticulocyte count of 2.55%. After 48 hours of phototherapy, the bilirubin gradually decreased to 13.6 mg/dL. However, upon cessation of phototherapy, the bilirubin level rose to 15.2 mg/dL, prompting resumption of phototherapy.

**Figure 5:**
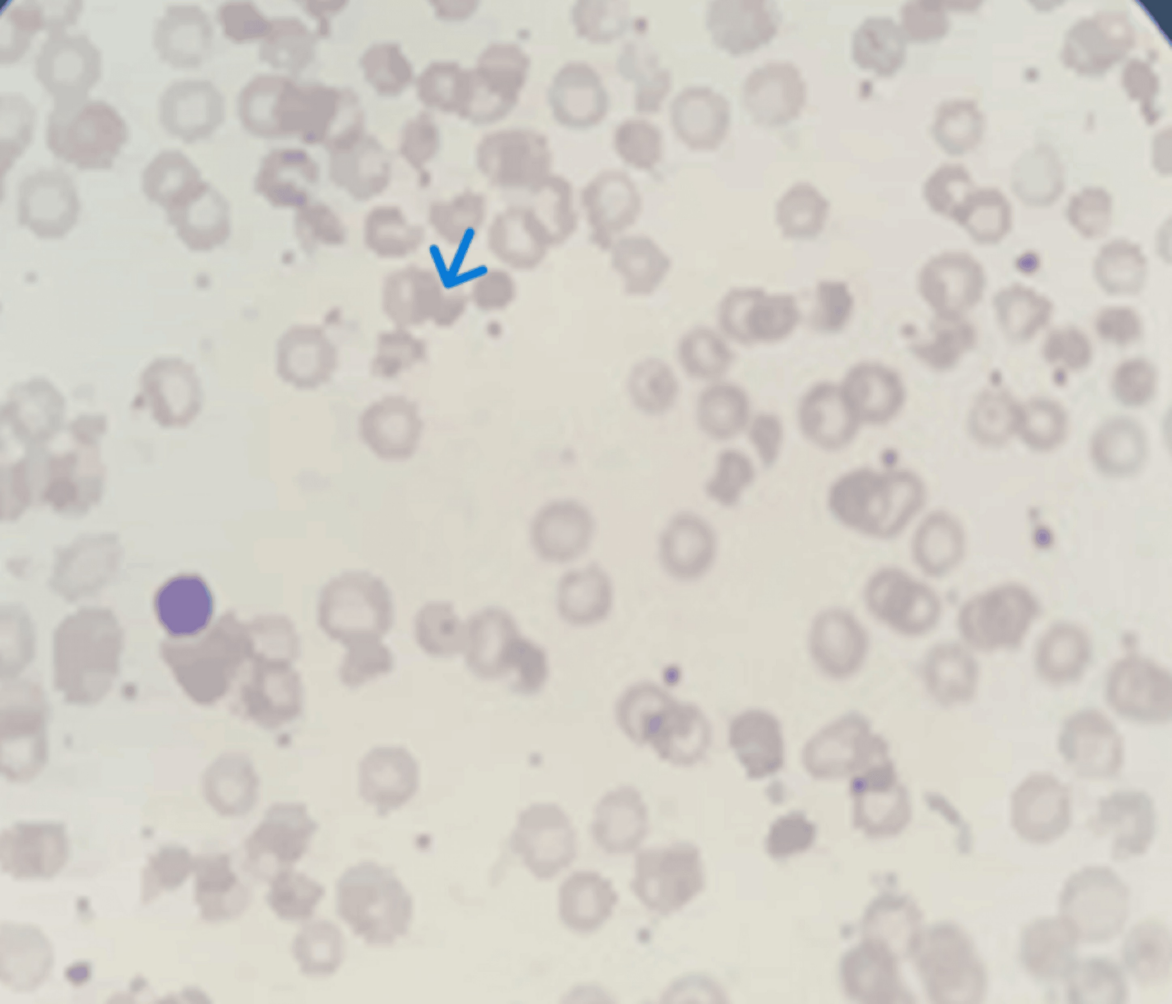
Blood film of case 2. The blood film reveals normocytic normochromic anemia with the presence of bitten RBCs, echinocytes, polychromatic cells, schistocytes, blister cells, and occasional spherocytes. Blue arrows mark pyknocytes, supporting the diagnosis of infantile pyknocytosis. WBCs show relative lymphocytosis with reactive forms, and platelets are elevated.

Concurrently, the baby’s hemoglobin level declined to 6.3 g/dL, with an increased reticulocyte count of 9.19%. Blood film analysis indicated acute hemolysis with irregular red cell morphology, suggestive of infantile pyknocytosis (Figure 5). A PRBC transfusion was administered to address the low hemoglobin level, resulting in an improvement to 13.2 g/dL. Subsequent response to phototherapy led to a reduction in bilirubin level to 13.4 mg/dL before discharge.

Additionally, a cardiac murmur was detected, and echocardiography revealed a 4 mm atrial septal defect (ASD II), for which follow-up at six months of age was advised. Regrettably, due to missed appointments, no further hemoglobin reports were obtainable.

Case 3

A preterm boy, born at 33 weeks of gestation, with a history of five days in the NICU (Neonatal Intensive Care Unit) for respiratory distress, presented to our facility at 25 days of age with a week’s history of jaundice that had intensified over the past three days. During his NICU stay, the baby had jaundice with high bilirubin levels for his age and received phototherapy, which was discontinued once bilirubin levels normalized. Both the mother and the neonate had blood group O positive. The parents are cousins but have no significant history of risk factors for hemolytic anemia.

**Figure 6:**
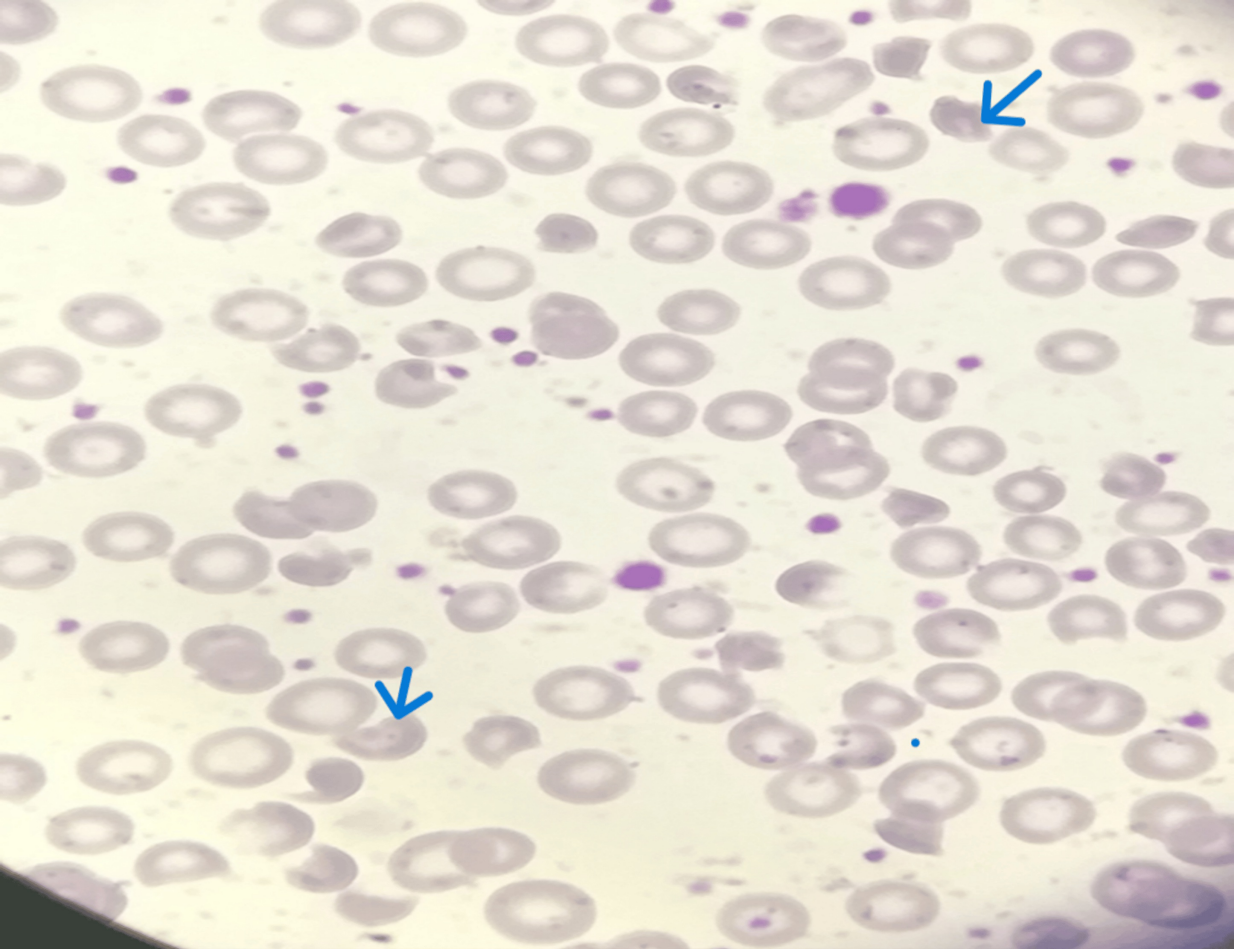
Blood film of case 3. The smear demonstrates normochromic normocytic RBCs with polychromatic macrocytes, normoblasts, schistocytes, and irregular contracted cells. Pyknocytes are indicated by blue arrows. Additional findings include bitten cells, spherocytes, and microspherocytes. WBCs show reactive lymphocytes; platelets are adequate.

On the 25th day of life, the baby was brought back due to yellowish discoloration of the skin and sclera, with parental concerns about reduced activity. Otherwise, the baby was vitally and clinically stable, with no documented fevers or sources of infection, feeding well on combination feeds of breast and formula milk, and passing normally colored urine and stool. Upon admission, the baby’s bilirubin level was 20.8 mg/dL, with an indirect bilirubin measurement of 19.6 mg/dL. A complete blood count (CBC) revealed RBC 2.78 × 10^6^/μL, Hb 9.6 g/dL, and a reticulocyte count of 11.12%. G6PD and pyruvate kinase levels were within normal ranges, and the Coombs test was negative. Additionally, the blood film indicated irregular erythrocyte morphology suggestive of infantile pyknocytosis (Figure 6).

Post-admission, he received intensive phototherapy and intravenous fluids. Bilirubin levels were closely monitored, and phototherapy was discontinued once levels normalized. Upon discharge, his serum bilirubin had decreased to 14.7 mg/dL; however, his hemoglobin had dropped to 7.9 g/dL, with a reticulocyte count of 17%. Subsequent blood tests one month later showed a hemoglobin level of 10 g/dL, and follow-up at three years of age revealed a hemoglobin level of 11.6 g/dL.

Case 4

A female term newborn, sibling of case 3, was brought in for a bilirubin check on the fifth day of life. Her bilirubin level was found to be 19.4 mg/dL, necessitating intensive phototherapy. Upon admission, her hemoglobin level was 17.1 g/dL, with a reticulocyte count of 3.8%. As phototherapy continued, her bilirubin level decreased to 7.7 mg/dL, at which point phototherapy was stopped. However, her bilirubin level quickly rose to 17.4 mg/dL within a few hours of discontinuing phototherapy, so the treatment was restarted.

**Figure 7:**
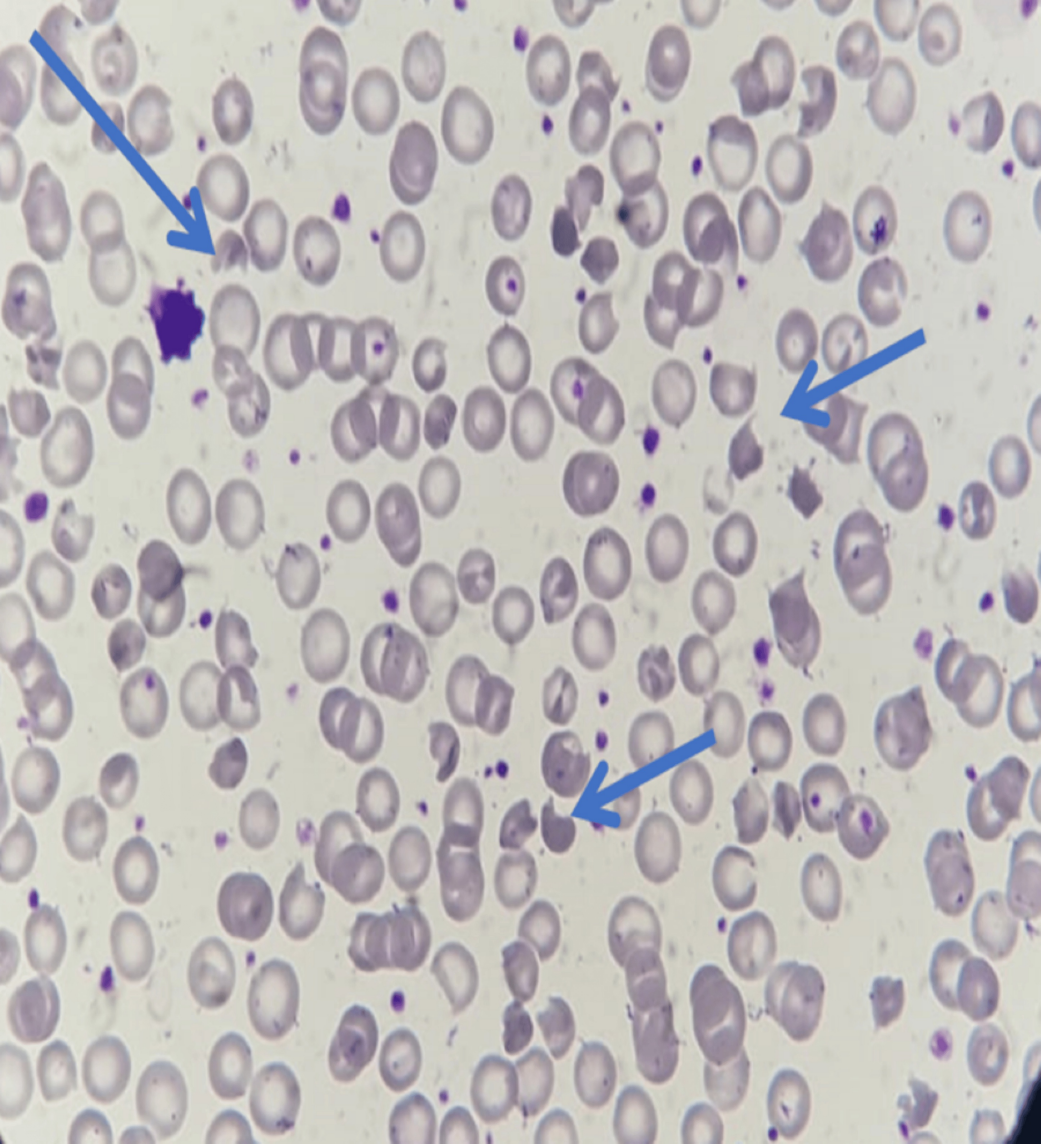
Blood film of case 4. Moderate normocytic normochromic anemia is evident with irregular contracted RBCs, bite cells, and normoblasts. Pyknocytes, marked by blue arrows, are consistent with the diagnosis of infantile pyknocytosis.

Due to persistent jaundice, a full blood count (FBC) was repeated seven days after admission, revealing a drop in hemoglobin from 17.1 to 10.6 g/dL, while the reticulocyte count remained at 2.3%. A blood film showed moderate normocytic normochromic anemia with irregularly contracted red blood cells, bite cells, and normoblasts, consistent with a diagnosis of infantile pyknocytosis, similar to her brother (Figure 7).

Hemoglobin was closely monitored, and on the 14th day of life, it dropped to 7.6 g/dL, prompting a PRBC transfusion, after which it increased to 9.7 g/dL. The baby was discharged after improvements in hemoglobin and bilirubin, with bilirubin falling below the threshold for phototherapy.

The day after discharge, the infant was reviewed at another facility, where her hemoglobin had improved to 11.1 g/dL and bilirubin was 14.3 mg/dL. Unfortunately, there are no further reports on blood indices, as she missed follow-up appointments.

## Discussion

Infantile pyknocytosis is a poorly understood cause of hemolytic anemia in newborns, resulting in prolonged jaundice and acute hemolysis that gradually resolves on its own without recurrence beyond the infantile period [6]. As a diagnosis of exclusion, only a few cases have been reported in the literature so far [7]. Despite the prevalence of other hemolytic anemias such as sickle cell anemia and thalassemia in the Middle Eastern population, infantile pyknocytosis remains an entity of scarcity of reports within the region [8,9].

Etiology

The etiology of infantile pyknocytosis remains largely unknown. While genetic factors have not been definitively established, some case reports suggest a potential familial tendency, as observed in siblings diagnosed with infantile pyknocytosis [10]. This is particularly relevant to the last two cases discussed in our series, where the neonates were born to first-degree consanguineous parents. However, comprehensive genetic studies are still lacking to confirm specific hereditary patterns.

Pathophysiology

Although the exact pathophysiology of infantile pyknocytosis remains poorly understood, there is a hypothesis involving extracorpuscular factors linked to oxidative stress and cellular damage. The conversion of transfused red blood cells into pyknocytes during exchange transfusions, as observed by Ackerman et al., suggests that external factors can induce changes in red blood cell morphology [11]. Furthermore, in a cohort study of 16 patients with infantile pyknocytosis, Heinz bodies were evident in 50% of the newborns but in 100% of cases after prolonged incubation of four hours. Heinz bodies are rounded, dark, or basophilic inclusions of denatured hemoglobin attached to the membrane, indicating oxidative damage. Although Heinz bodies can occasionally be found in healthy neonates, their presence in 100% of cases correlates with the involvement of oxidative stress in infantile pyknocytosis [12]. In our case series, two patients developed hemolysis and jaundice with blood films suggestive of infantile pyknocytosis in the context of respiratory and urinary tract infections, indicating that infectious pathogens could exacerbate oxidative damage to red blood cells.

Despite the hypothesis that extrinsic factors contribute to hemolysis, infantile pyknocytosis is characterized by Coombs-negative hemolytic anemia, indicating that the hemolysis is not immune-mediated, such as that seen with ABO or Rh incompatibility. This suggests that intrinsic factors could affect red blood cell stability. Potential intrinsic mechanisms include membrane instability, whereby inherent defects in the red blood cell membrane may lead to increased fragility and susceptibility to hemolysis, and metabolic deficiencies. Although not definitively linked to infantile pyknocytosis, deficiencies in enzymes responsible for maintaining red blood cell integrity or membrane protective factors could contribute to hemolytic processes. For example, a case report by Clare Rees et al. described reduced levels of the glutathione peroxidase enzyme in the absence of a mutation in the gene [13]. Glutathione peroxidases are a family of enzymes that catalyze the reduction of hydroperoxides with varying substrate and electron donor specificities. Since their discovery, glutathione peroxidases have been associated with protecting RBCs from oxidative damage; therefore, a deficiency of the enzyme could generate damage to the lipid membrane [14].

The pathophysiology of infantile pyknocytosis appears to involve a complex interaction between extrinsic factors, such as infections, and various elements contributing to oxidative stress. Additionally, intrinsic factors that influence red blood cell membrane stability or provide protection against oxidative damage play a significant role. While further research is essential to fully elucidate these mechanisms, the current understanding underscores the necessity for early identification, continuous monitoring, and effective management of affected infants.

Diagnosis

Diagnosing infantile pyknocytosis presents challenges due to its classification as a diagnosis of exclusion. The primary diagnostic indicators include clinical signs such as jaundice occurring within the first week of life, which can be prolonged, recurrent, and severe enough to require phototherapy, as illustrated in our case series. Additionally, symptoms of hemolytic anemia, such as pallor and lethargy due to significant anemia, are critical for diagnosis.

Laboratory findings are essential, particularly the identification of more than 5% pyknocytes, which are irregularly shaped red blood cells with short projections, on peripheral blood smears. Blood film morphology typically reveals features indicative of hemolysis, including polychromatic macrocytes, normoblasts, schistocytes, bite cells, echinocytes, blister cells, occasional spherocytes, and target cells. Furthermore, a negative DAT is instrumental in ruling out autoimmune causes of hemolytic anemia, thereby differentiating infantile pyknocytosis from conditions such as ABO or Rh incompatibilities or isoimmune hemolytic diseases [15].

Infantile pyknocytosis and congenital heart defects

In addition to the known clinical features suggestive of infantile pyknocytosis (jaundice and anemia), our case series revealed that two neonates (cases 1 and 2) exhibited cardiac murmurs upon examination. Subsequent echocardiograms identified structural heart defects: a large patent ductus arteriosus (PDA) with a bidirectional shunt in case 1 and an atrial septal defect (ASD) with a 4 mm defect in both cases.

Although ASDs occur in 1.6 per thousand live births [16], the presence of cardiac defects in infants with infantile pyknocytosis may not be coincidental. While the literature does not establish a direct causal relationship between infantile pyknocytosis and congenital heart defects, their occurrence in the same patient could suggest shared risk factors or developmental anomalies during gestation. Some congenital heart defects have genetic components, and a familial predisposition to either infantile pyknocytosis or cardiac anomalies could explain their co-occurrence in affected neonates.

Additionally, the anemia associated with infantile pyknocytosis can lead to increased cardiac workload due to compensatory mechanisms such as elevated heart rate and stroke volume. This may exacerbate underlying cardiac conditions or reveal previously undiagnosed heart defects. While infantile pyknocytosis is primarily a hematological disorder, its association with cardiac defects in some cases highlights the importance of comprehensive evaluations in affected neonates, particularly if they present with signs of respiratory distress or other symptoms indicating cardiovascular burden. Further research is needed to explore potential links between these two conditions, including shared etiological factors or genetic predispositions.

Infantile pyknocytosis and pulmonary hypertension

In the first case reported in our series, the infant developed key features of pulmonary hypertension with increased pulmonary arterial pressure on an echocardiogram (ECHO) as early as four weeks of age. This could be attributed to two potential explanations.

Cardiac Complications

The congenital heart defects observed in case 1, such as a large patent ductus arteriosus (PDA), atrial septal defect (ASD), and tricuspid regurgitation, could lead to increased workload on the right ventricle. If left untreated or inadequately managed, this may contribute to pulmonary vascular changes over time.

Systemic Stress Response

Severe anemia requiring multiple transfusions can result in systemic inflammation and oxidative stress responses. A case reported by Hanane et al. demonstrated the presence of pulmonary hypertension in an 11-day-old neonate with severe hemolytic anemia, supporting the hypothesis that pulmonary hypertension may be secondary to or aggravated by neonatal hemolysis [17]. Pulmonary hypertension resulting from hematological disorders can arise from pre-capillary causes, post-capillary causes, or a combination of both. The underlying mechanisms may involve hemolysis and its effects, chronic anemia leading to elevated cardiac output, or a hypercoagulable state.

Nitric oxide (NO) plays a crucial role as a potent vasodilator, regulator of endothelial cell proliferation, and anti-inflammatory agent. A reduction in nitric oxide and its precursor, arginine, has been linked to an increased incidence of pulmonary hypertension. Intravascular hemolysis releases free hemoglobin and arginase-1, both of which diminish nitric oxide signaling through different mechanisms. Free hemoglobin directly inactivates nitric oxide, while arginase-1 reduces the availability of L-arginine, a substrate necessary for nitric oxide synthase. These processes lead to decreased nitric oxide levels and impaired vascular endothelial function, potentially resulting in pre-capillary pulmonary hypertension. Beyond hemolysis, chronic anemia can create a high cardiac output state that contributes to left-sided heart disease and left ventricular dysfunction, increasing the risk of post-capillary pulmonary hypertension. Furthermore, hemolysis induces oxidative damage to tissues, activates platelets, and triggers the coagulation cascade, resulting in vasculopathy and hypercoagulability. Consequently, patients face a heightened risk of developing deep venous thrombosis and pulmonary embolism, which are significant contributors to pulmonary hypertension [18].

Although the onset of pulmonary hypertension can be rapid, significant intravascular hemolysis may play a role in either initiating or exacerbating pulmonary hypertension. This situation prompts consideration of whether infants diagnosed with severe hemolytic anemia should be screened for pulmonary hypertension.

Management

Management of infantile pyknocytosis primarily involves supportive care. Phototherapy was effectively utilized in all cases to manage hyperbilirubinemia, while PRBC transfusions were necessary for those with significant anemia. The use of recombinant human erythropoietin has shown promise in some studies as an adjunct therapy for managing anemia in these patients. Literature suggests that early intervention can lead to favorable outcomes; however, ongoing monitoring is essential to ensure that bilirubin levels decrease appropriately and that hemoglobin levels stabilize [19]. In this series, all neonates showed improvement post-transfusion and phototherapy, reinforcing the importance of timely medical intervention.

Prognosis 

As highlighted in the majority of published literature, the prognosis for infants with this condition is generally positive, as it is often self-limiting and tends to resolve spontaneously within four to six months. In our case series, follow-up of hemoglobin levels in case 1 and case 3 demonstrated natural resolution of the condition, with subsequent visits confirming the expected prognosis. Follow-up studies indicate no evidence of symptom recurrence beyond the infantile stage. Infants typically return to normal health without any long-term complications related to the condition. For example, follow-up evaluations at three years of age for case 3 showed normal hemoglobin levels and overall health in previously affected infants, with no signs of recurrence.

Complications

While infantile pyknocytosis is usually self-limiting with appropriate treatment, failing to address the condition can lead to serious complications. These may include kernicterus due to prolonged jaundice and elevated bilirubin levels, which can result in long-term neurological deficits. Severe anemia can also impose cardiovascular strain, potentially leading to complications such as pulmonary hypertension or heart failure, particularly in infants with pre-existing conditions or anatomical heart defects. This was illustrated in the first case of our series and is supported by Hanane et al., who reported mortality associated with pulmonary hypertension [17].

## Conclusions

Infantile pyknocytosis represents an under-recognized cause of neonatal hemolytic anemia that warrants attention in clinical practice. The presented case series underscores the necessity for vigilance in diagnosing this condition amidst more prevalent hemolytic disorders. With appropriate recognition and management, infants diagnosed with infantile pyknocytosis can expect a favorable prognosis without lasting effects. Future research should focus on elucidating the pathophysiology of infantile pyknocytosis and on establishing standardized diagnostic criteria to aid clinicians in identifying this rare condition more effectively.

## References

[1] Aher S, Malwatkar K, Kadam S (2008). Neonatal anemia. Semin Fetal Neonatal Med.

[2] Nassin ML, Lapping-Carr G, de Jong JL (2015). Anemia in the neonate: the differential diagnosis and treatment. Pediatr Ann.

[3] Tuffy P, Brown AK, Zuelzer WW (1959). Infantile pyknocytosis; a common erythrocyte abnormality of the first trimester. AMA J Dis Child.

[4] Eyssette-Guerreau S, Bader-Meunier B, Garcon L, Guitton C, Cynober T (2006). Infantile pyknocytosis: a cause of haemolytic anaemia of the newborn. Br J Haematol.

[5] Evangelos C, Konstantina A, Theodora B, Varvara D, Mirsini M, Dimitris D (2020). Infantile pyknocytosis: a rare cause of newborn hemolytic anemia - two case reports. Pediatr Hematol Oncol J.

[6] (2023). UFO Themes. Red cell disorders. https://oncohemakey.com/red-cell-disorders/.

[7] Drouilly M, Jourdan L, Gérard D (2024). Infantile pyknocytosis, a neonatal hemolytic anemia with Heinz bodies: A cohort study. Pediatr Blood Cancer.

[8] Kavanagh PL, Fasipe TA, Wun T (2022). Sickle cell disease: a review. JAMA.

[9] Harteveld CL, Higgs DR (2010). Alpha-thalassaemia. Orphanet J Rare Dis.

[10] Maxwell DJ, Seshadri R, Rumpf DJ, Miller JM (1983). Infantile pyknocytosis: a cause of intrauterine haemolysis in 2 siblings. Aust N Z J Obstet Gynaecol.

[11] Ackerman BD (1969). Infantile pyknocytosis in Mexican-American infants. Am J Dis Child.

[12] Saeidian H (2024). Infantile pyknocytosis: the presence of abnormal erythrocytes in newborn and infants. Clin Pediatr Open Access.

[13] Rees C, Lund K, Bain BJ (2019). Infantile pyknocytosis. Am J Hematol.

[14] Orrico F, Laurance S, Lopez AC (2023). Oxidative stress in healthy and pathological red blood cells. Biomolecules.

[15] Kraus D, Yacobovich J, Hoffer V, Scheuerman O, Tamary H, Garty BZ (2010). Infantile pyknocytosis: a rare form of neonatal anemia. Isr Med Assoc J.

[16] Menillo AM, Lee L, Pearson-Shaver AL (2023). Atrial septal defect (ASD). StatPearls [Internet].

[17] Dahoui HA, Abboud MR, Saab R, Farra C, Sinno D, Dabbous I, Muwakkit SA (2008). Familial infantile pyknocytosis in association with pulmonary hypertension. Pediatr Blood Cancer.

[18] Haw A, Palevsky HI (2018). Pulmonary hypertension in chronic hemolytic anemias: pathophysiology and treatment. Respir Med.

[19] Buzzi E, Scognamillo R, Girardi E, Amendola G, Dall’Agnola A (2013). Infantile pyknocytosis: effectiveness of erythropoietin treatment. J Pediatr Neonat Individual Med.

